# PEDRo: A database for storing, searching and disseminating experimental proteomics data

**DOI:** 10.1186/1471-2164-5-68

**Published:** 2004-09-17

**Authors:** Kevin Garwood, Thomas McLaughlin, Chris Garwood, Scott Joens, Norman Morrison, Christopher F Taylor, Kathleen Carroll, Caroline Evans, Anthony D Whetton, Sarah Hart, David Stead, Zhikang Yin, Alistair JP Brown, Andrew Hesketh, Keith Chater, Lena Hansson, Muriel Mewissen, Peter Ghazal, Julie Howard, Kathryn S Lilley, Simon J Gaskell, Andy Brass, Simon J Hubbard, Stephen G Oliver, Norman W Paton

**Affiliations:** 1 Department of Computer Science, University of Manchester, Oxford Road, Manchester M13 9PL, UK; 2 School of Biological Sciences, University of Manchester, Oxford Road, Manchester M13 9PT, UK; 3 European Bioinformatics Institute, Wellcome Trust Genome Campus, Hinxton, Cambridgeshire CB10 1SD, UK; 4 School of Biomolecular Sciences, UMIST, PO Box 88, Manchester M60 1QD, UK; 5 School of Medical Sciences, Institute of Medical Sciences, University of Aberdeen, Aberdeen AB25 2ZD, UK; 6 Department of Molecular Microbiology, John Innes Centre, Norwich Research Park, Colney, Norwich NR4 7UH, UK; 7 Scottish Centre for Genomic Technology & Informatics, University of Edinburgh Medical School, The Chancellor's Building, Little France Crescent, Edinburgh, EH16 4S, UK; 8 University of Cambridge, Department of Biochemistry, Downing Site, Cambridge, CB2 1QW, UK; 9 Department of Chemistry, UMIST, PO Box 88, Manchester M60 1QD, UK

## Abstract

**Background:**

Proteomics is rapidly evolving into a high-throughput technology, in which substantial and systematic studies are conducted on samples from a wide range of physiological, developmental, or pathological conditions. Reference maps from 2D gels are widely circulated. However, there is, as yet, no formally accepted standard representation to support the sharing of proteomics data, and little systematic dissemination of comprehensive proteomic data sets.

**Results:**

This paper describes the design, implementation and use of a Proteome Experimental Data Repository (PEDRo), which makes comprehensive proteomics data sets available for browsing, searching and downloading. It is also serves to extend the debate on the level of detail at which proteomics data should be captured, the sorts of facilities that should be provided by proteome data management systems, and the techniques by which such facilities can be made available.

**Conclusions:**

The PEDRo database provides access to a collection of comprehensive descriptions of experimental data sets in proteomics. Not only are these data sets interesting in and of themselves, they also provide a useful early validation of the PEDRo data model, which has served as a starting point for the ongoing standardisation activity through the Proteome Standards Initiative of the Human Proteome Organisation.

## Background

Bioinformatics tools and techniques depend directly or indirectly upon experimental data. However, interpreting experimental data often requires access to significant amounts of additional information about the sample used in the experiment, the conditions in which measurements were taken, the equipment used to take the measurements, etc. Recent proposals for models that capture such experimental descriptions alongside experimental results include MIAME for transcriptome data [1] and PEDRo for proteome data [2]. However, if full use is to be made of such rich data models for genomic data, these models must also be associated with comprehensive software tools for data capture, dissemination and analysis.

In proteomics, which is rapidly evolving into a high-throughput experimental approach, there is (as yet) no standard representation for experimental data. As a result, limited tool support is available for disseminating, searching, comparing or analysing the results of experiments conducted using different techniques and equipment in different laboratories. Thus, while experimental results can be analysed, often in a labour-intensive manner in-house, the development of bioinformatics techniques for archiving, sharing and wider exploitation of proteomics results is still in its infancy. This paper seeks to contribute to the development of effective and systematic support for proteome data management by:

1. Describing a database for storing, searching and disseminating experimental proteomics data. This material should be relevant to the developers of future proteome data management systems in that it discusses and illustrates various design and implementation decisions that have an impact on the role and maintenance of the resulting database.

2. Making available data sets from several labs whose data have been included in the initial release of the database. These data sets themselves result from substantial experimental activities, and are representative of the sorts of information that in-house and public proteome data repositories must capture. As the database stores data in an XML format that conforms to the PEDRo (Proteomics Experimental Data Repository) data model [2], this material provides concrete examples for other users of data that conform to this schema, and should be useful for validation of specific parts of the model as input to the Human Proteome Organisation Proteome Standards Initiative (HUPO-PSI) activity on models for proteome data [3].

The database described in this paper has similar objectives and functionality to various other databases for functional genomic data. In particular, like the Gene Expression Omnibus (GEO) [4], the Stanford Microarray Database [5] and ArrayExpress [6], it contains a single category of experimental data, while accommodating the production of that data using several different experimental techniques. Like ArrayExpress, and unlike GEO, for example, the data stored in PEDRo must conform to a rich, but nevertheless deliberately constraining, data model. This model is richer than that supported by the well established SWISS-2DPAGE database [7] in that it not only contains information on protein separation and identification, but also includes detailed descriptions of experimental samples, the mass spectrometric analyses conducted, and the software used to perform protein identifications.

Establishing the most appropriate kinds of data to include in a database such as PEDRo is not straightforward, as this depends on the use that is to be made of the data. In a large data repository, users may want to search for results based on widely varying criteria – for example, the proteins identified, the change in the level of a protein over time, the mechanism by which a sample was studied, etc. Furthermore, the users of a proteome data repository may themselves be diverse, and include: experimentalists with minimal direct experience of proteomics, but who are interested in proteins or organisms for which proteome studies have been conducted; proteome scientists who want to identify how successful specific techniques have been in different contexts; or mass-spectrometric analysts who want to compare their results with those of others.

This wide range of potential users encourages the creation of a rich repository for proteomics data that provides detailed descriptions of many different aspects of an experiment. However, populating a database such as PEDRo is not a trivial task, as several of the different kinds of data included in PEDRo currently have to be entered manually, which is time-consuming for data providers. Even though a data entry tool has been developed to ease data entry (available from [8]), experience populating the database suggests that the creation of a data set from scratch (e.g., for a sample analysed using a single gel, for which multiple identifications have taken place) can take around a week, but that creating subsequent data sets that share some aspects of the experimental set-up is significantly less time-consuming. In addition, widespread deployment of a standard model should lead to laboratory equipment, or associated software, producing data that conforms to the standard, so the longer-term position for high-throughput laboratories should involve much lower data capture costs. It is hoped that the early provision of a collection of data sets conforming to the widely discussed PEDRo model will be useful in informing ongoing activities on the HUPO-PSI proteome data standard [3].

## Construction and content

Many bioinformatics databases, such as UniProt [9] and PDB [10], are associated with file formats that can be parsed by software that analyses or displays the data from the database. The Extensible Markup Language (XML [11]) has been developed in part to make the description, parsing and display of such files more systematic; thus there is a trend in bioinformatics towards the use of XML for storing or transmitting biological data [1,2].

**Figure 1:**
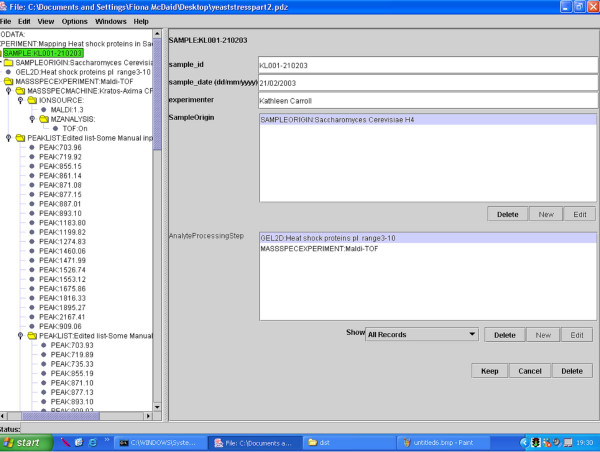
**The Pedro data capture tool. **The Pedro data capture tool in use, editing a proteome data file. The left hand panel provides a tree-based browser for the complete document, while the right hand panel supports data entry for a specific component of the model, in this case a *sample*. The data capture tool is available for download from [8].

**Figure 2:**
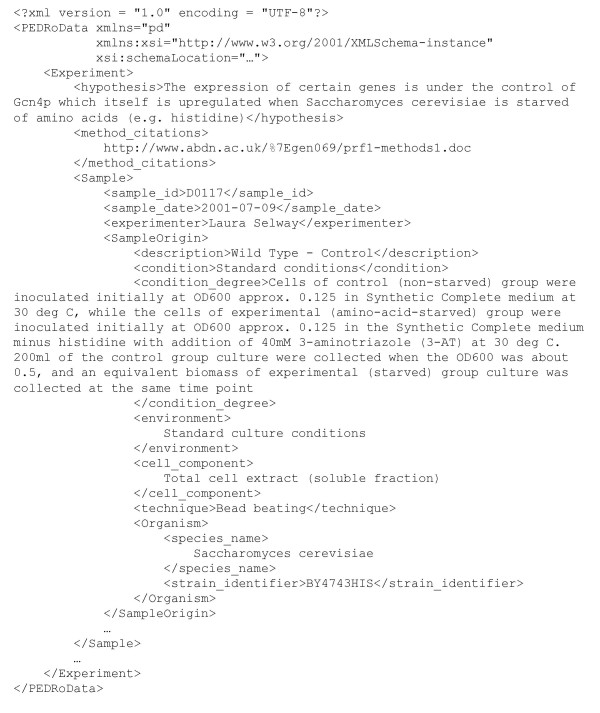
**Sample PEDRo XML. **A fragment of XML for an *S. cerevisiae *sample. The XML Schema from the model, plus complete data sets for the experiments described in Table 1, are available from [8].

The PEDRo database makes extensive use of XML for capturing, transmitting, storing and searching proteomics data. In particular:

1. The data-capture process uses a software tool, illustrated in Figure 1, which prompts users for values for different fields, and includes facilities for importing substantial data files, such as those representing peak lists. The tool constructs data-entry forms from the XML Schema definition of the PEDRo model. An XML Schema describes the structure of an XML document, and thus makes explicit the hierarchical structure of the document, the elements that are contained within the document, the types of those elements, and the number of times different elements may occur. The result of the data capture process is thus an XML file that corresponds to the PEDRo schema. A fragment of the XML format for a PEDRo entry is provided in Figure 2.

2. The database stores the XML captured using the data entry tool directly, using Xindice [12], an open-source XML storage system. Several different storage options exist for XML data, including: (i) storing the XML directly in a native XML repository such as Xindice; (ii) storing the XML directly using the XML storage extensions provided by commercial relational database vendors; and (iii) mapping from the XML documents onto tables for storage in a relational database. We have chosen option (i) for PEDRo. Option (ii) was not adopted because there is not yet a standard for integrating XML storage with relational databases, although this is being developed [13]. Option (iii) was not adopted because we envisage that the data model used in PEDRo will evolve to reflect the HUPO-PSI standard [3], and we wanted to avoid the need to evolve both relational and XML versions of the database in parallel. Furthermore, the emphasis for the PEDRo database is on enabling users to identify relevant experimental data sets, rather than on conducting complex searches or analyses over such data sets. For the required tasks, the query facilities provided with XML databases such as Xindice, which tend for the meantime to be based upon XPath [14] are sufficient.

3. The data are presented to users by generating web pages from the stored XML using XSLT [15], which was designed to support exactly this sort of task. This means that it has been straightforward to develop reports from the stored form of the data. Furthermore, the download format for the data is as XML documents, in the hope that this will ease the development of tools for parsing and analysing data obtained from the database.

**Figure 3:**
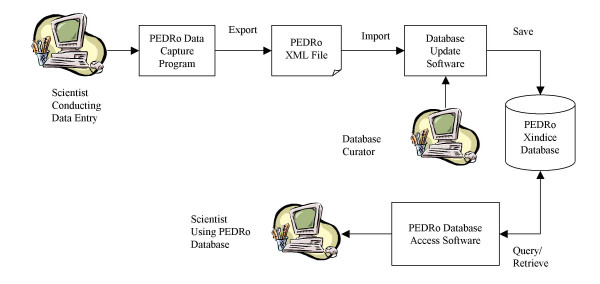
**PEDRo software components. **The software components used in PEDRo. In essence, data flows clockwise from the top left, with three categories of user. The first category of user is the scientist who carries out data entry – this user must be intimately familiar with the experiment that has been conducted and the equipment that has been used. The result of the data capture process is a PEDRo XML file. This XML file is then checked by the database curator, who can then add the data into the database. Once in the database, the PEDRo Database Access software can be used to search the database and view its contents. The PEDRo software has been implemented over the Xindice XML database [12] using Java Server Pages [28].

The software components used within PEDRo (and the role they play in data capture, storage and dissemination) are illustrated in Figure 3.

The data stored in the database for each experiment is as described in the PEDRo model [2], and thus involves *sample generation *(e.g. organism, growth conditions, tagging), *sample processing *(e.g. gel properties, spot details), *mass spectrometry *(e.g. machine settings, peak lists) and *in silico analyses *(e.g. database search program used and results obtained). Table 1 provides a high-level overview of the initial data sets in the database. The data in the initial release of the database illustrates several different proteomics techniques in use, including sample processing based on classical 2D Gels and DIGE, the use of different gel imaging software, mass spectrometry using MALDI-TOF and MS/MS, and *in silico *data analyses using more than one program. Furthermore, the data captured covers a range of different organisms, including *Saccharomyces cerevisiae*, *Candida albicans*, *Candida glabrata*, *Mus musculus*, *Arabidopsis thaliana *and *Streptomyces coelicolor*.

**Table 1:** Summary of database contents. A summary of the data sets included in the initial release of PEDRo. The database provides more detailed descriptions of *sample generation*, *sample processing*, *mass spectrometry *and *in silico analyses*, populating the model described in [2].

**1**	**Organism**	*Saccharomyces cerevisiae*	**Sample Generation**	Whole cell extracts (bead beating)
	**Experimenter**	Al Brown *et al.*	**Sample Processing**	2D gel
	**Description**	GCN4-dependent proteins that respond to amino acid starvation	**Mass Spectrometry**	MALDI-ToF
			***In Silico*Analysis**	Peptide mass fingerprint searches (MASCOT, MS-Fit)
**2**	**Organism**	*Candida albicans*	**Sample Generation**	Whole cell extracts (bead beating)
	**Experimenter**	Al Brown *et al.*	**Sample Processing**	2D gel
	**Description**	GCN4-dependent proteins that respond to amino acid starvation	**Mass Spectrometry**	MALDI-ToF
			***In Silico*Analysis**	Peptide mass fingerprint searches (MASCOT, MS-Fit)
**3**	**Organism**	*Candida glabrata*	**Sample Generation**	Whole cell extracts (bead beating)
	**Experimenter**	Ken Haynes *et al.*	**Sample Processing**	2D gel
	**Description**	Proteins that respond to the inactivation of ACE2	**Mass Spectrometry**	MALDI-ToF
			***In Silico*Analysis**	Peptide mass fingerprint searches (MASCOT, MS-Fit)
**4**	**Organism**	*Streptomyces coelicolor*	**Sample Generation**	Whole cell extracts (sonication)
	**Experimenter**	Andrew Hesketh	**Sample Processing**	2D gel
	**Description**	Changes in the proteome of strain M600 during growth and antibiotic production in liquid medium	**Mass Spectrometry**	MALDI-ToF
			***In Silico*Analysis**	Peptide mass fingerprint searches (MASCOT)
**5**	**Organism**	*Mus Musculus BALB/C*	**Sample Generation**	Exfoliated jejunal epithelium prepared as per Bjerknes & Cheng, Anat Rec 1981, 199, 565.
	**Experimenter**	Alan Pemberton, Pamela Knight	**Sample Processing**	2D gel
	**Description**	Trichinella spiralis infection in mice induces alterations in the proteome of the small mucosal epithelium	**Mass Spectrometry**	MALDI-ToF
			***In Silico*Analysis**	Peptide mass fingerprint searches (MASCOT, MS-FIT)
**6**	**Organism**	*Homo sapiens*	**Sample Generation**	Gel image analysis data from 12 patients, used to assign spot picks from a preparative 2D gel
	**Experimenter**	Tony Whetton, Caroline Evans	**Sample Processing**	2D gel
	**Description**	Proteomics can identify prognostic markers in CLL disease progression	**Mass Spectrometry**	MALDI-ToF
			***In Silico*Analysis**	Peptide mass fingerprint searches (MASCOT)
**7**	**Organism**	*Saccharomyces cerevisiae*	**Sample Generation**	*Saccharomyces cerevisiae*, strain YMK36, butanol – and butanol +
	**Experimenter**	Kathleen Carroll	**Sample Processing**	2D gel
	**Description**	Effect of butanol stress on yeast	**Mass Spectrometry**	MALDI-ToF
			***In Silico*Analysis**	Peptide mass fingerprint searches (MASCOT)
**8**	**Organism**	*Saccharomyces cerevisiae*	**Sample Generation**	
	**Experimenter**	Kathleen Carroll	**Sample Processing**	2D gel
	**Description**	Mapping Heat shock proteins in *Saccharomyces cerevisiae*	**Mass Spectrometry**	MALDI-ToF
			***In Silico*Analysis**	Peptide mass fingerprint searches (MASCOT)
**9**	**Organism**	*T. bruceii*	**Sample Generation**	Gel pieces
	**Experimenter**	Sarah Hart	**Sample Processing**	Bands from 1D gel
	**Description**	Trypanosome Flagella	**Mass Spectrometry**	MALDI-ToF, LC MS/MS
			***In Silico*Analysis**	Mascot MS/MS

These PEDRo data are significant in biological terms. For example, they include the first direct comparison of proteomic responses in two fungal species, namely responses to amino-acid starvation in the baker's yeast *S. cerevisiae *and the pathogenic fungus, *C. albicans *[16]. In addition, they include the first proteomic analysis of the medically important pathogen *C. glabrata *(Stead *et al*., Proteomic changes associated with inactivation of the *Candida glabrata ACE2 *virulence-moderating gene, manuscript submitted), whose genome sequence has only just been completed. The *Streptomyces coelicolor M600 *data set is the largest proteomics time course analysis of this strain in terms of numbers of proteins identified. It adds significantly to our knowledge of expression of some of the 20 gene sets annotated as being determinants of the biosynthesis of secondary metabolites, including antibiotics. Somewhat similar experiments, but differing in many aspects of their metadata, are reported on the SWICZ database [17]. This provides an opportunity to evaluate PEDRo in the context of related data presented in different databases. Also included are data from an experiment investigating the proteomic analysis of the mouse jejunal epithelium and its response to infection with the intestinal nematode, *Trichinella spiralis *[18].

## Utility

Web-based interfaces to biological databases tend to support one or more of the following tasks: *browsing *– interactively listing or navigating through database entries; *searching *– identifying database entries on the basis of simple restrictions on the values of one or more fields; *visualising *– presenting a visual representation of the data as a starting point for browsing; or *querying *– specifying a search that is to be conducted over the database using a query building interface or by providing inputs to pre-written (or "canned") queries. Functional genomics databases tend to emphasise browsing and searching. For example, the Stanford Microarray Database [5] supports browsing based around organisms and experiments, and more complex Boolean searches based on criteria such as experimenter, organism and category of experiment. ArrayExpress [6] supports browsing through experiments, arrays and protocols, and searching based on criteria such as species, experiment type and author. SWISS-2DPAGE [7] supports browsing by clicking on spots on gels, and searching based on criteria such as description, accession number or author.

**Figure 4:**
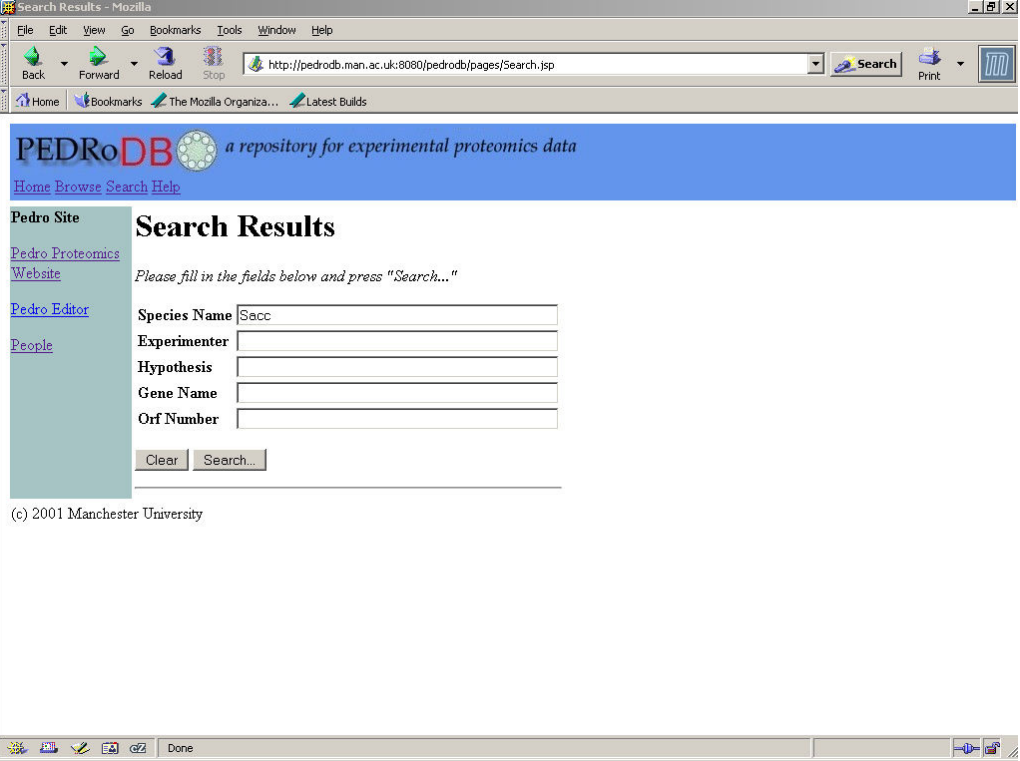
**Search page. **Searching the PEDRo database. The search facility is intended to support rapid identification of PEDRo entries of interest. Searching can be conducted on one or more of the species name, experimenter, hypothesis, gene name and ORF number. Where more than one value is provided, entries are retrieved that match all the values given. In all cases, matches can be partial. Thus, for example, typing "Sacc" into the *Species Name *field will retrieve entries for *Saccharomyces cerevisiae*.

**Figure 5:**
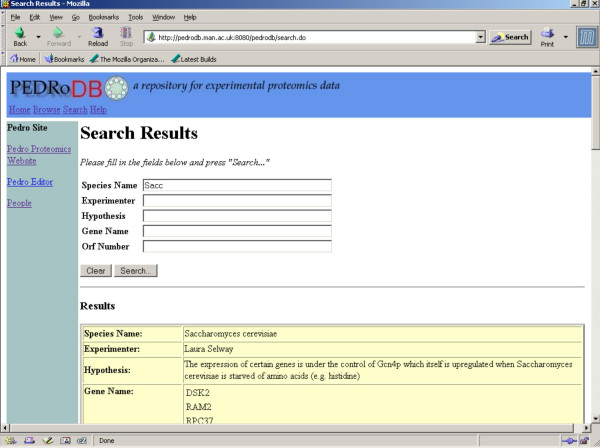
**Results page. **Viewing results of the PEDRo search from Figure 4.

PEDRo also emphasises browsing and searching. Figures 4 and 5 illustrate the web-based interface to PEDRo, which can be accessed at [8]. In essence, the records in the database can be accessed by browsing summaries of the entries in the database, or by searching using one or more criteria. These criteria were obtained through a systematic requirements analysis with potential users from several different research groups, who were asked to comment on early versions of the interface. Overall, PEDRo provides core data access facilities that are principally intended to allow users to identify data sets that are of interest to them. As such, the PEDRo database as described should not be seen as a comprehensive query or analysis environment for proteomics data, but rather as a repository through which experimental results can be made available to a wider community. Therefore, *S. cerevisiae *data from PEDRo will also be made available through GIMS [19], for example, to enable the integration of these data with other sequence and functional information.

## Discussion

A significant motivating factor behind the development of the PEDRo repository has been to allow informed discussion, assisted by concrete examples, into the level of detail and forms of model that are most appropriate for a proteome data repository. As the PEDRo model is being used as the starting point for the HUPO-PSI activity on models for proteome data, early validation of this model is important. The following observations have been made about the PEDRo model during the data capture process:

1. *Sample description is neither very precise nor systematic*. The effective description of samples is an open issue that spans different kinds of functional genomic data. For example, work is underway on the development of an ontology for characterising microarray experiments, focusing, in particular, on samples [20]. However, as the variety of organisms, genetic manipulations, extraction techniques, environmental conditions and experimental manipulations that may characterise a sample are extremely large, a mature solution to this problem may be some way off.

2. *There is only limited support for relative protein abundance data (e.g. DIGE and stable isotope labelling strategies)*. Thus, for example, there is no place in the model to describe an expression ratio for a protein species derived from quantitative experimental strategies, only the ability to capture the 'raw' numbers. In fact, the PEDRo model was not designed to capture expression ratios, partly because such numbers are easily derived from the captured primary data, and partly because the particular method of their derivation may be contentious. It is hoped that the HUPO-PSI model will provide generic constructs for representing relationships between certain kinds of measurement (e.g. relative protein expression readings), to which can be attached the specific detail for individual techniques. However, it also seems important to avoid the pitfalls associated with overly permissive models, as these provide a less stable foundation for the developers of analytical tools than their more proscriptive counterparts.

3. *The gel model is not particularly detailed*. Thus, for example, there is no detailed description of the image analysis software used, the descriptions of individual spots are fairly minimal, and no details are captured on spot excision. An earlier critique of the PEDRo model for gels, and some possible extensions, is provided by [21]. It seems that, in order to provide insights for the developers of gel-based experiments, it would be appropriate for the model to be revised to provide additional details on gels.

Overall, the appropriate level of detail for a proteomics repository is somewhat subjective, but can usefully be based on guiding principles; agreement as to the principles should then avoid scope-based discussions at a very fine-grained level. The current PEDRo model essentially supports the principle that enough detail should be captured about an experiment to:

i. Allow results of different experiments to be analysed/compared.

ii. Allow suitability of experiment design and implementation decisions to be assessed.

iii. Allow protein identifications to be re-run in the future with new databases or software.

There is also an additional negative principle, to the effect that the model itself should not be designed to include dependencies on characteristics relating to the configuration or properties of an individual piece of equipment. Accordingly, we have attempted to allow experimental methods and results to be described in significant detail, but without including parameters and properties that are likely to be superseded rapidly when new models of equipment are introduced, and without including parameters that can only be understood with reference to the documentation of a particular product.

The data stored in PEDRo is more comprehensive for each experiment than is the case for most existing proteome databases. For example, in the longest established experimental proteomics resource, SWISS-2DPAGE [7], the emphasis is on annotated gels, and there is much less information collected on how the annotations were arrived at. Furthermore, there is an architectural distinction – SWISS-2DPAGE follows a more federated approach, with individual sites continuing to hold their own data. These other proteome data sources can be accessed through WORLD-2DPAGE, a web resource listing sites making available experimental proteomics data [22]. An example of a database that participates in WORLD-2DPAGE is the University of Alabama (UAB) Proteomics Database [23] which provides search and browsing facilities over data from its host university. As such, the emphasis is on annotated gels, and relatively few details are captured on sample processing, mass spectrometry or *in silico *analysis. Such design decisions are appropriate for certain categories of user of a proteomics database, but not for others. The UAB database has been designed to provide access to processed experimental results for biomedical researchers, but does not provide enough information to allow detailed comparisons of the ways in which the results were obtained.

The ProteomeWeb [24] provides a wider range of tools than PEDRo (for example, for computing theoretical maps), and supports browsing of annotated gels from several bacteria and archaea. Once again, though, the data provided for each experiment are less comprehensive than in PEDRo. ProDB [25] has a certain amount in common with UAB, in that it too provides search and browsing over a database of locally produced data. In addition, ProDB features an architecture that supports the plugging-in of data-loading and analysis tools. However, the level of detail supported by the model is not obvious from the paper, which gives only part of the model, and the database was not publicly accessible at the time of writing. In consisting of a collection of tools associated with a database, ProDB thus also has a certain amount in common with SBEAMS [26] which includes a relational database of proteomic data. The SBEAMS model emphasises the description and analysis of mass spectrometry data, but seems not to support open access to experimental data at the time of writing.

In terms of quantities of data, there are fewer data sets in PEDRo than in SWISS-2DPAGE, reflecting the fact that PEDRo is a newly created resource (Release 16 of SWISS-2DPAGE contains 34 reference maps), but somewhat more than in the UAB Proteomics Database. The Open Proteomics Database (OPD) supports the browsing and downloading of comparable amounts of data to those in PEDRo, and also includes mass spectrometry data, although quite a lot of the data are in flat-file format [27]. However, it is fair to say that none of the current databases is operating in the context of high-throughput experimentation, which will certainly be prevalent in the near future.

## Conclusions

The need for wider and more systematic dissemination of experimental proteomics data is widely recognised, as argued in [27], and attested to by the ongoing work of the Proteome Standards Initiative [3]. As such, issues that need to be addressed include:

i. The nature and variety of information that should be recorded about proteomics experiments.

ii. The functionality that should be provided by repositories that make large-scale proteomic data available.

iii. The computational architecture that should be used to provide the functionality at (ii).

iv. The nature of the tools that should be developed for use with such a repository.

This paper has sought to address issues (i), (ii) and (iii), with a particular emphasis on (i). Following on from [2] we believe that the provision of a collection of representative proteomic data sets conforming to a consistent model is important to the ongoing process of developing a stable and effective *de jure *standard for proteome data representation and sharing. This paper describes a database that includes a rich collection of representative data sets. Furthermore, the paper describes the functionality (issue ii) and architecture (issue iii) of an exploratory system for disseminating such data. In the same way as we see models for representing proteomic data evolving in the light of practical experience, we anticipate that the PEDRo repository, and the overall understanding of the data access and dissemination requirements for proteomic data, will evolve as the opportunities presented by high-throughput experimental techniques and comprehensive data sets become more fully understood.

## Availability and requirements

The database can be accessed using a web browser at , by following the *Database *link.

## Authors' contributions

KG and CG implemented the software. SJ, NM and CFT contributed to the development of earlier prototypes. TM coordinated the data capture activity. CK, CE, AW, SH, DS, ZY, AJPB, AH, KC, LH, MM, PG, JH, KSL, SJG conducted or led experimental activities that generated the data in the database, and contributed to feedback on the model. AB, SJH, SJO and NWP oversaw the database design and development activity, and the latter led the write-up.

## References

[B1] Spellman PT, Miller M, Stewart J, Troup C, Sarkans U, Chervitz S, Bernhart D, Sherlock G, Ball C, Lepage M, wiatek M, Marks WL, Goncalves J, Markel S, Iordan D, Shojatalab M, Pizarro A, White J, Hubley R, Deutsch E, Senger M, Aronow BJ, Robinson A, Bassett D, Stoeckert CJ, Brazma A (2002). Design and implementation of microarray gene expression markup language (MAGE-ML). Genome Biol.

[B2] Taylor CF, Paton NW, Garwood KL, Kirby PD, Stead DA, Yin ZK, Deutsch EW, Selway L, Walker J, Riba-Garcia I, Mohammed S, Deery MJ, Howard JA, Dunkley T, Aebersold R, Kell DB, Lilley KS, Roepstorff P, Yates JR, Brass A, Brown AJP, Cash P, Gaskell SJ, Hubbard SJ, Oliver SG (2003). A systematic approach to modeling, capturing, and disseminating proteomics experimental data. Nature Biotech.

[B3] Orchard S, Zu W, Julian RK, Hermjakob H, Apweiler R (2003). Further advances in the development of a data interchange standard for proteomics data. Proteomics.

[B4] Edgar R, Domrachev M, Lash AE (2002). Gene Expression Omnibus: NCBI gene expression and hybridisation array data repository,. Nucleic Acids Research.

[B5] Gollub J, Ball CA, Binkley G, Demeter J, Finkelstein DB, Hebert JM, Hernandez-Boussard T, Jin H, Kaloper M, Matese JC, Schroeder M, Brown PO, Botstein D (2003). The Stanford Microarray Database: data access and quality assessment tools,. Nucleic Acids Research.

[B6] Brazma A, Parkinson H, Sarkans U, Shojatalab M, Vilo J, Abeygunawardena N, Holloway E, Kapushesky M, Kemmeren P, Lara GG, Oezcimen A, Rocca-Serra P, Sansone S-A (2003). ArrayExpress – a public repository for microarray gene expression data at the EBI,. Nucleic Acids Research.

[B7] Hoogland C, Sanchez J-C, Tonella L, Binz P-A, Bairoch A, Hochstrasser DF, Appel RD (2000). The 1999 SWISS-2DPAGE database Update,. Nucleic Acids Research.

[B8] PEDRo Web Site. http://pedro.man.ac.uk.

[B9] Apweiler R, Bairoch A, Wu CH, Barker WC, Boeckmann B, Ferro S, Gasteiger E, Huang H, Lopez R, Magrane M, Martin MJ, Natale DA, O'Donovan C, Redaschi N, Yeh LS (2004). UniProt: the Universal Protein Knowledgebase,. Nucleic Acids Res.

[B10] Berman HM, Westbrook J, Feng Z, Gilliland G, Bhat TN, Weissig H, Shindyalov IN, Bourne PE The Protein Databank,. Nucleic Acids Research.

[B11] Extensible Markup Language. http://www.w3.org/XML.

[B12] Xindice. http://xml.apache.org/xindice/.

[B13] Eisenberg A, Melton J (2002). SQL/XML is making good progress,. ACM SIGMOD Record.

[B14] XPath. http://www.w3.org/TR/xpath.

[B15] XSLT. http://www.w3.org/TR/xslt.

[B16] Yin Z, Stead D, Selway L, Walker J, Riba-Garcia I, Mclnerney T, Gaskell S, Oliver SG, Cash P, Brown AJP (2004). Divergence between *Candida albicans *and *Saccharomyces cerevisiae *in their globalresponses to amino acid starvation. Proteomics.

[B17] SWICZ. http://proteom.biomed.cas.cz.

[B18] Pemberton AD, Knight PA, Wright SH, Miller HRP (2004). Proteomic analysis of mouse jejunal epithelium and its response to infection with the intestinal nematode,. Proteomics.

[B19] Cornell M, Paton NW, Hedeler C, Kirby P, Delneri D, Hayes A, Oliver SG (2003). GIMS: An integrated data storage and analysis environment for genomic and functional data. Yeast.

[B20] Stoeckert C, Parkinson H (2003). The MGED ontology: a framework for describing functional genomics experiments. Comparative and Functional Genomics.

[B21] Jones A, Wastling J, Hunt E (2003). Proposal for a standard representation of two-dimensional gel electrophoresis data,. Comparative and Functional Genomics.

[B22] WORLD-2D-PAGE. http://us.expasy.org/ch2d/2d-index.html.

[B23] Hill A, Kim H (2003). The UAB Proteomics Database. Bioinformatics.

[B24] Babnigg G, Giometti CS (2003). ProteomeWeb: A web-based interface for the display and interrogation of proteomes. Proteomics.

[B25] Wilke A (2003). Bioinformatics support for high-throughput proteomics,. Journal of Biotechnology.

[B26] SBEAMS. http://www.sbeams.org/.

[B27] Prince JT, Carlson MW, Wang R, Lu P, Marcotte EM (2004). The need for a public proteomics repository,. Nature Biotec.

[B28] Java Server Pages. http://java.sun.com/products/jsp/.

